# Transcriptome-based analysis of blood samples reveals elevation of DNA damage response, neutrophil degranulation, cancer and neurodegenerative pathways in *Plasmodium falciparum* patients

**DOI:** 10.1186/s12936-021-03918-5

**Published:** 2021-09-26

**Authors:** Akua A. Karikari, Wasco Wruck, James Adjaye

**Affiliations:** 1 Department of Biomedical Sciences, College of Health and Allied Sciences, University of Cape Coast, Cape Coast, Ghana; 2 Institute for Stem Cell Research and Regenerative Medicine, Medical Faculty, Heinrich-Heine University, 40225 Düsseldorf, Germany

**Keywords:** Cerebral malaria, *P. falciparum*, Cellular stress, Immune response, Neutrophil degranulation, Axonal dysfunction

## Abstract

**Background:**

Malaria caused by *Plasmodium falciparum* results in severe complications including cerebral malaria (CM) especially in children. While the majority of falciparum malaria survivors make a full recovery, there are reports of some patients ending up with neurological sequelae or cognitive deficit.

**Methods:**

An analysis of pooled transcriptome data of whole blood samples derived from two studies involving various *P. falciparum* infections, comprising mild malaria (MM), non-cerebral severe malaria (NCM) and CM was performed. Pathways and gene ontologies (GOs) elevated in the distinct *P. falciparum* infections were determined.

**Results:**

In all, 2876 genes were expressed in common between the 3 forms of falciparum malaria, with CM having the least number of expressed genes. In contrast to other research findings, the analysis from this study showed MM share similar biological processes with cancer and neurodegenerative diseases, NCM is associated with drug resistance and glutathione metabolism and CM is correlated with endocannabinoid signalling and non-alcoholic fatty liver disease (NAFLD). GO revealed the terms biogenesis, DNA damage response and IL-10 production in MM, down-regulation of cytoskeletal organization and amyloid-beta clearance in NCM and aberrant signalling, neutrophil degranulation and gene repression in CM. Differential gene expression analysis between CM and NCM showed the up-regulation of neutrophil activation and response to herbicides, while regulation of axon diameter was down-regulated in CM.

**Conclusions:**

Results from this study reveal that *P. falciparum*-mediated inflammatory and cellular stress mechanisms may impair brain function in MM, NCM and CM. However, the neurological deficits predominantly reported in CM cases could be attributed to the down-regulation of various genes involved in cellular function through transcriptional repression, axonal dysfunction, dysregulation of signalling pathways and neurodegeneration. It is anticipated that the data from this study, might form the basis for future hypothesis-driven malaria research.

**Supplementary Information:**

The online version contains supplementary material available at 10.1186/s12936-021-03918-5.

## Background

Malaria is a major infectious disease and public health problem with an estimated 215 million cases and 384,000 deaths in 2019 [1]. The disease is most severe in Africa, where the World Health Organization (WHO) malaria report in 2020 revealed that the region accounts for 94% of malaria cases and deaths universally [1]. Of the five human parasite species, *Plasmodium falciparum* and *Plasmodium vivax* are the most common cause of severe malaria [2]. *Plasmodium falciparum* is however primarily implicated in cerebral malaria (CM) in children under 5 years of age and pregnant women [3–5]. It is also resistant to many anti-malarial drugs making it more lethal.

The life cycle of the apicomplexan P. *falciparum* within the human host includes the intra-erythrocytic stage where haem is produced from the host’s haemoglobin [6]. *Plasmodium falciparum* also harbours the “plant-like” apicoplast, which is essential for parasite de novo synthesis of haem as a back-up [7, 8]. Haem is used by the parasite mitochondrial electron transport chain and its toxicity is attenuated by cross-linking into an insoluble polymer (haemozoin), via the action of a parasite-specific biochemical activity [6, 9, 10]. Haemozoin (HMZ), also classified as malaria pigment, is typically observed in the liver, spleen and brain of infected individuals [11, 12]. The severity of falciparum malaria correlates with the release of HMZ, which can be incorporated into membranes and also diffuse through the blood brain barrier (BBB) [3, 13–15]. Though *P. falciparum* does not invade the brain parenchyma, toxic HMZ crosses the BBB, causing injuries to neurons [16, 17]. Germane to HMZ pathology is the production of reactive oxygen species (ROS), which is implicated in oxidative macromolecular damage, inflammatory response, endoplasmic reticulum (ER) stress and apoptosis [18–22]. The developing brain is especially susceptible to genomic instability and cellular stress engendered by oxidative modification of macromolecules and this is also linked to neurodevelopmental and neurodegenerative diseases, including schizophrenia, Alzheimer’s (AD) and Parkinson’s (PD) diseases [23–27]. Interestingly, reports by Thiam et al*.* and Cabantous et al*.* demonstrated the activation of genes associated with AD and PD in CM [28, 29]. In addition, Boldt et al*.* found a signature of 22 differentially expressed genes related to immunopathological processes and complement regulation in the transcriptome of distinct conditions of childhood malaria including CM [30].

In this study, an analysis of two pooled series of microarray of transcriptome data of whole blood cells derived from patients with mild malaria (MM), non-cerebral severe malaria (NCM) and CM were carried out. The results showed that fewer genes are expressed in CM compared to the other states of malaria. Additionally, genes involved in neutrophil degranulation and response to herbicides were up-regulated in CM, whereas genes associated with axon diameter were down-regulated. Genes connected to DNA repair mechanisms and cytoskeletal destabilization were significantly expressed in MM and NCM, respectively.

Thus, the neurological impairment, such as cognitive decline reported in falciparum malaria patients, especially CM, could be attributed to inflammation, genomic instability and cellular stress-mediated neuronal malfunctioning and degeneration.

## Methods

### Preprocessing of gene expression data for the pooled analysis

Datasets of the accession numbers GSE1124 and GSE116306 containing transcriptome microarray data of cerebral malaria, non-cerebral severe malaria and mild malaria were downloaded from NCBI GEO. For GSE1124, children within the ages of 0.5–6 years were used whereas patients within ages of 1–72 years were employed in GSE116306. The datasets employed in this study are listed in Table 1. GSE1124 was associated with a publication by Boldt et al*.* [30] and GSE116306 was associated with Thiam et al*.* [28]. The number of subjects involved in the study were 4–6 (MM:6, NCM:4 and CM:6) for Thiam et al*.* [28] and 20 samples per each group for Boldt et al. [30]. Thiam et al*.* [28] classified MM as fever with *P. falciparum* parasitaemia of < 25,000 parasites/μl of blood, with no proof of severe malaria characteristics, NCM as severe anaemia, respiratory distress or hypoxia and hypoglycaemia without neurological symptoms and CM as absence of purposeful response, presence of a deep coma, deficit in response to a painful stimulus by Glasgow score < 9. For Boldt et al. [30], the definition of MM was presence of *P. falciparum* parasitaemia by microscopic examination of thick and thin blood smears with no evidence of other severe diseases, NCM was haemoglobin level < 5 g/dL and CM was Blantyre coma score ≤ 2. A complete blood count was conducted in both studies and Boldt et al*.* [30] report that the experimental groups differed significantly with respect to age, respiration rate, degree of spleen enlargement, white blood cell count, glycaemia, haemoglobin and haematocrit levels. In contrast, Thiam et al*.* [28] stated that with the exception of platelet count, which differed between the experimental groups, there was no significant difference between the groups for age, haemoglobin concentration, red blood cell count and leucocyte count. For comparison of distinct disease states including CM, Boldt et al*.* had processed their data with proprietary software followed by application of Significance Analysis of Microarrays (SAM) on the logarithmic (base 2) signal ratios to identify significantly changed genes at a false discovery rate of 0.004%. The datasets from the accession GSE1124, which were generated on the *Affymetrix Human Genome U133A Array* platform, were used. Datasets from the accession GSE116306 were generated on the *Agilent-039494 SurePrint G3 Human GE v2 8* × *60 K Microarray* platform. All datasets were imported into the R/Bioconductor environment [31].

**Table 1:** Datasets employed in this pooled analysis

GEO_sample	Sample name	Group	GEO_accession
GSM3227838	CM1	CM	GSE116306
GSM3227839	CM2	CM	GSE116306
GSM3227840	CM3	CM	GSE116306
GSM3227841	CM4	CM	GSE116306
GSM3227842	CM5	CM	GSE116306
GSM3227843	CM6	CM	GSE116306
GSM3227844	MM1	MM	GSE116306
GSM3227845	MM2	MM	GSE116306
GSM3227846	MM3	MM	GSE116306
GSM3227847	MM4	MM	GSE116306
GSM3227848	MM5	MM	GSE116306
GSM3227849	MM6	MM	GSE116306
GSM3227850	NCM1	NCM	GSE116306
GSM3227851	NCM2	NCM	GSE116306
GSM3227852	NCM3	NCM	GSE116306
GSM3227853	NCM4	NCM	GSE116306
GSM711624	Asymptomatic_A	Asymptomatic	GSE1124
GSM711625	Asymptomatic_B	Asymptomatic	GSE1124
GSM711626	Asymptomatic_C	Asymptomatic	GSE1124
GSM711627	Asymptomatic_F	Asymptomatic	GSE1124
GSM711628	Asymptomatic_G	Asymptomatic	GSE1124
GSM711629	Uncomplicated_malaria_D	MM	GSE1124
GSM711630	Uncomplicated_malaria_E	MM	GSE1124
GSM711631	Uncomplicated_malaria_F	MM	GSE1124
GSM711632	Uncomplicated_malaria_G	MM	GSE1124
GSM711633	Uncomplicated_malaria_H	MM	GSE1124
GSM711634	Severe_malaria_A	NCM	GSE1124
GSM711635	Severe_malaria_B	NCM	GSE1124
GSM711636	Severe_malaria_C	NCM	GSE1124
GSM711637	Severe_malaria_D	NCM	GSE1124
GSM711638	Severe_malaria_E	NCM	GSE1124
GSM711639	Cerebral_malaria_C	CM	GSE1124
GSM711640	Cerebral_malaria_D	CM	GSE1124
GSM711641	Cerebral_malaria_E	CM	GSE1124
GSM711642	Cerebral_malaria_F	CM	GSE1124
GSM711643	Cerebral_malaria_G	CM	GSE1124

*MM* mild malaria, *NCM* severe non-cerebral malaria, *CM* cerebral malaria. Datasets from the accession GSE1124 are associated with Boldt et al., datasets from the accession GSE116306 are associated with Thiam et al

Furthermore, the datasets generated on the Agilent platform were read and preprocessed employing the Bioconductor package limma [32]. This included background correction with the method normexp and quantile normalization. The binary score “gIsWellAboveBG” from the input data files was used as binary measure for gene expression. Multiple probes matching the same gene symbol were reduced to the probe with the maximal mean expression signal.

Datasets generated on the Affymetrix platform were read and pre-processed employing the Bioconductor package affy [33]. This included background correction and normalization with the Robust Multi-Array average (RMA) method. Background detection values were calculated via the *mas5calls* method[33]. Analogously to the other platform multiple probes matching the same gene symbol were reduced to the probe with the maximal mean expression signal.

### Analysis of pooled gene expression data

The preprocessed datasets from the GEO accessions GSE1124 and GSE116306 were adjusted for batch effects via the ComBat method [34] from the Bioconductor package sva [35]. In the follow-up analyses this batch-effect-adjusted data was used for differential expression and cluster analysis while the binary expression score described above was used for the Venn diagram analysis. The global cluster dendrogram was generated via the R method *hclust* from the log2-transformed expression data with a coefficient of variation above 0.1 employing Pearson correlation as similarity measure and complete linkage as agglomeration method. Differentially expressed genes were determined with the criteria ratio > 2 and p-value < 0.05 and q-value < 0.25 for up- and ratio < 0.5 and p-value < 0.05 and q-value < 0.25 for the down-regulated genes (the distribution of ratios is shown in Additional file 1). The p-value was calculated with the method from the Bioconductor package limma [32]. The p-value was adjusted for multiple-testing with the package qvalue [36]. The heatmaps were generated with the method heatmap.2 from the package gplots [37]. For the Venn diagram analysis genes were considered expressed when the mean of the binary expression score (1 if expressed, else 0) was above a threshold of 0.5 in the Agilent data and the mean of the Affymetrix detection-p-values was below 0.05 in the distinct disease states. The Venn diagrams were generated with the package VennDiagram [38].

### Over-representation analysis of pathways and gene ontologies (GOs)

Pathways annotated with gene symbols were downloaded from the Kyoto Encyclopedia of Genes and Genomes (KEGG) database [39] in July 2020. Genes found in subsets of the Venn diagram analysis and differential gene expression analysis were tested for over-representation in these gene sets via the hypergeometric distribution test implemented in R. Furthermore, these subsets of genes were analysed for over-representation in GOs with the R package GOstats [40]. Dot plots of pathways or GOs most significantly elevated were generated via the R package ggplot2 [41] (Additional file 1).

### Metascape analysis of differentially expressed genes

Lists of genes differentially up- (ratio > 2, limma-p-value < 0.05, q-value < 0.25) and down-regulated (ratio < 0.5, limma-p-value < 0.05, q-value < 0.25) between CM and NCM were joined to a two-column-list, which was subjected to Metscape multi-column-list analysis [42]. From the results the Protein–protein-interaction (PPI) network and the heatmap of regulating transcription factors generated via the TRRUST method [43] were used for this publication.

## Results

The pathways and gene expression variations of the pooled microarray datasets of whole blood cells derived from children and adult patients suffering from MM, NCM and CM are represented as dendrograms, Venn diagrams and heatmaps.

### Cluster analysis of CM in comparison to MM and NCM

**Fig. 1:**
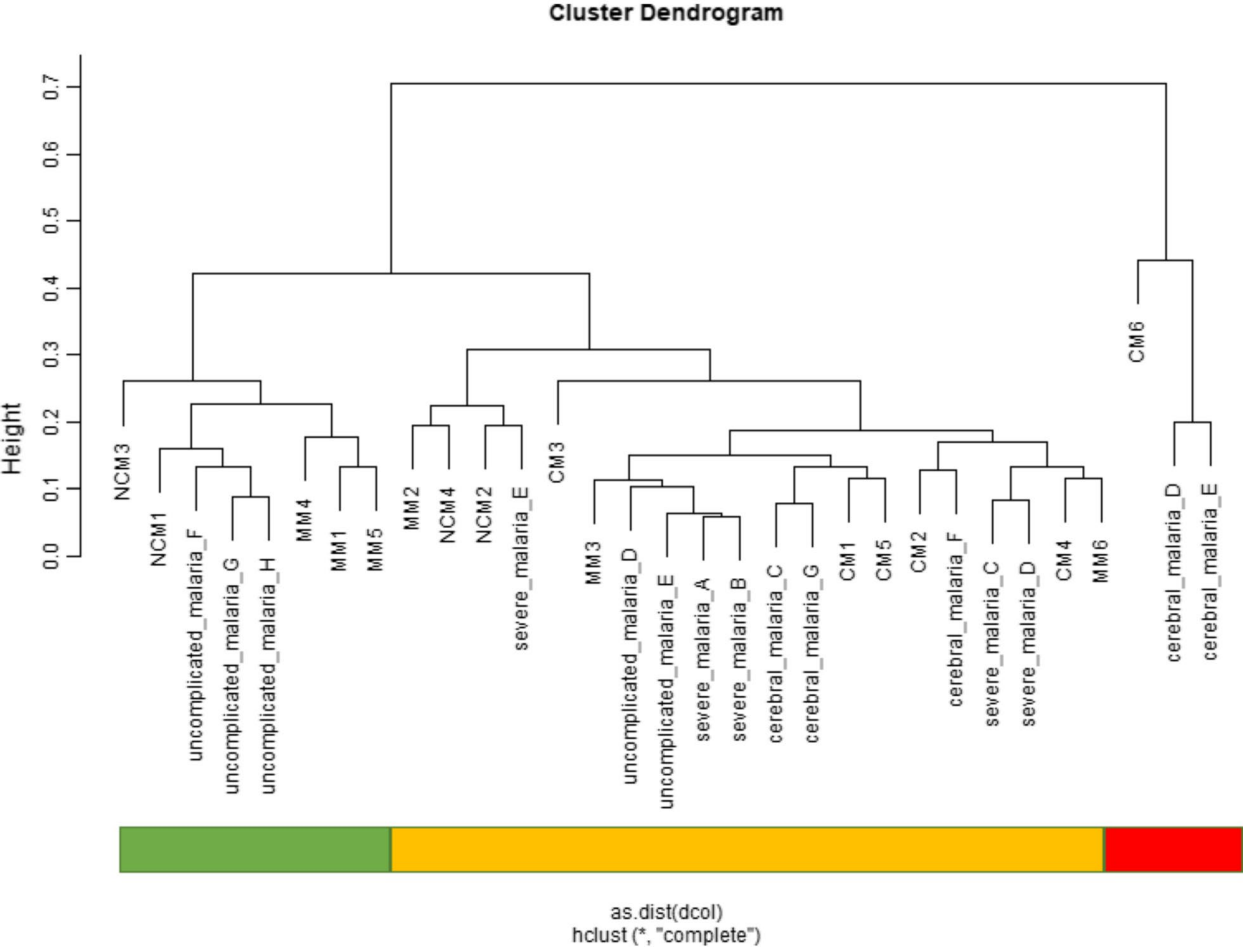
Cluster analysis dendrogram of malaria patient’s transcriptomes displays three clusters with varying severity. The cluster to the right is consistently CM, however contains only three samples, the cluster to the left is more mild, the one in the middle more severe. The distinct clusters are marked with colour bars: cluster 1 (green, predominantly mild), cluster 2 (orange, predominantly severe and cerebral), cluster 3 (red, consistently CM). *CM* cerebral malaria, *MM* mild malaria and *NCM* severe non-cerebral malaria

Figure 1 shows a categorized cluster of the datasets used in the pooled analysis namely, GSE116306 and GSE1124. In GSE116306, MM corresponds to “mild malaria”, NCM is “severe non-cerebral malaria” and CM refers to “cerebral malaria”. For GSE1124, MM stands for “uncomplicated malaria”, NCM refers to “severe malaria” and CM is “cerebral malaria”. The cluster dendrogram indicates that while there is a general overlap between the different states of falciparum malaria, CM cases predominantly share characteristics with NCM when compared to MM.

### Fewer genes are expressed in CM

**Fig. 2:**
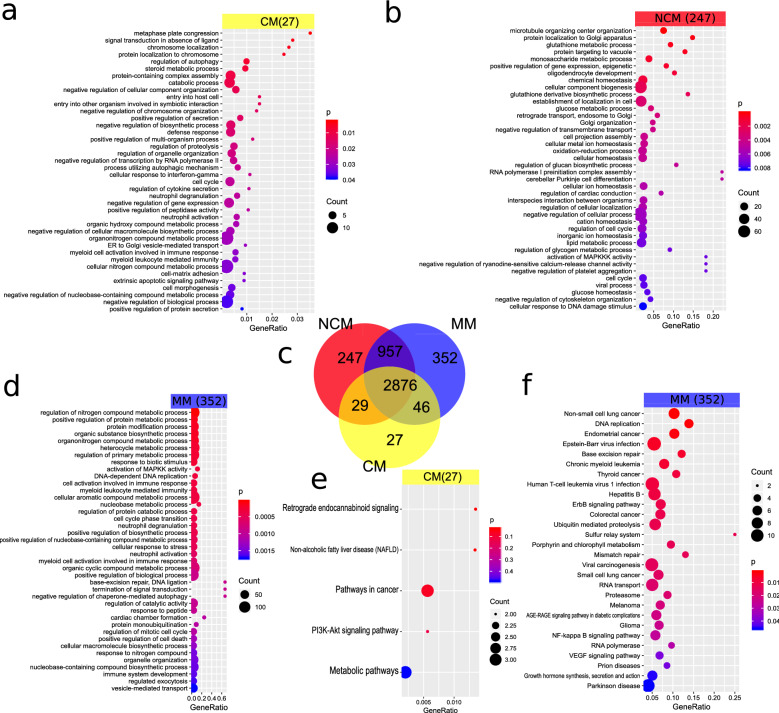
Comparison of gene expression between CM, NCM and MM shows endocannabinoid signalling in CM, neurodegenerative pathways with MM and drug resistance pathways were associated with NCM. Panels **a**, **b** and **d** show GO expression analysis results and **e** and **f** show pathway analysis results of the genes expressed exclusively in CM, NCM or MM in the Venn diagram in (c). 27 genes are expressed exclusively in CM while in the NCM there are 247 genes and in MM 352 genes exclusively expressed. Most genes (2876) are expressed in common in all disease states. **a** The GO analysis reveals signal transduction abnormality, neutrophil degranulation and negative regulation of gene expression amongst the most significantly expressed GO terms in the 27 CM genes. **b** Amongst the significant GO terms for the 247 genes expressed exclusively in NCM were *microtubule organizing center organization, glutathione metabolic process, glutathione derivative biosynthetic process, oxidation–reduction process, DNA damage checkpoint, mitotic cell cycle arrest, reactive oxygen species biosynthetic process, removal of superoxide radicals, detoxification, activation of mitogen-activated protein* kinases *(MAP3Ks) activity, negative regulation of cytoskeleton organization and amyloid-beta clearance.* These terms are suggestive of glutathione involvement in defense against ROS in severe malaria, DNA damage repair mechanisms, cytoskeletal destabilization activation and protein clearance. **d** Elevated GO terms for the 352 genes exclusively expressed in MM included *biogenesis*, *regulation of nitrogen compound metabolic process, positive regulation of protein metabolic process, response to biotic stimulus, DNA damage response, detection of DNA damage, positive regulation of phospholipid metabolic process*, *blood vessel remodeling, cell cycle arrest, response to unfolded protein, immune response* and *interleukin (IL)-10 production*, which are indicative of DNA repair mechanisms, cellular repair and anti-inflammatory mechanisms. **e** Over-represented KEGG pathways for the CM-exclusive genes included *retrograde endocannabinoid signalling* and **f** over-represented KEGG pathways for the MM-exclusive genes included *non-small cell lung cancer*, *endometrial cancer*, *Epstein-Barr virus (EBV) infection, base excision repair* and *mismatch repair*-which are pointers to DNA damage and increased risk of cancer development—and also pathways for *Parkinson’s* and *prion disease* thus reflecting distinct neurodegenerative gene expression patterns in MM

Gene expression was analysed between MM, NCM and CM and represented in a Venn diagram in Fig. 2c. In all, there were 2876 genes mutually expressed between the three disease states. There were 352 and 247 genes independently expressed in MM and NCM, respectively. In CM (27 genes) however, several genes were down-regulated which included those associated with cytoskeletal organization, translation, cellular transport, cellular respiration, mitochondrial functioning and proteasomal processes. Genes expressed in common between the 3 classes of malaria comprised those of inflammatory response (CKLF), ER stress (RNF13), detoxification of toxic metals and antioxidant response (MT1X), cellular stress (HSPs), clathrin and adaptor protein complex 2 assembly (PICALM) and mental retardation (AUTS2). It is noteworthy that genes implicated in AD, (PSEN1 and APP), were also up-regulated in MM, NCM and CM.

### Cancer, DNA repair and PD pathways are associated with MM

To identify pathways associated with the different states of malaria, GO and KEGG pathway enrichment analyses were conducted. The most significant pathways that correlated with the 352 genes specific to MM (Fig. 2f, Additional file 2: Table S1g) were *Non-small cell lung cancer* (p < 0.0007), *Endometrial cancer* (0.0016), *Epstein-Barr virus (EBV) infection* (0.0046), *DNA replication* (p < 0.0010), *Base excision repair* (0.0055) and *Mismatch repair* (p < 0.0132). These pathways are suggestive of damage to host DNA and the subsequent increased risk of cancer development. Interestingly, the pathways for *Parkinson’s disease* (PD) and *Prion disease* (PrD) were up-regulated (Fig. 2f, Additional file 2: Table S1g). This shows that MM though mild, could have deleterious implications on the brain.

### Drug resistance, glutathione metabolism and endocannabinoid signalling correlate with NCM and CM

The most significant pathways that were associated with NCM (Additional file 2: Table S1f) included *Platinum drug resistance* (p < 0.0025) *Nucleotide excision repair* (p < 0.0226) and *Glutathione metabolism* (p < 0.0373), which implies drug resistance, DNA damage and antioxidant processes during *P. falciparum* infection. With regards to CM (Fig. 2e, Additional file 2: Table S1e), pathways involving *Retrograde endocannabinoid signalling* (p < 0.0265) and *Non-alcoholic fatty liver disease (NAFLD)* (p < 0.0269) were most relevant. This could reflect an endocannabinoid modulation in CM pathology and hepatocyte injury.

### GO analysis of genes enriched in MM

Subsequently, an assessment of the GOs elevated in *P. falciparum* infection was conducted. Figure 2d and Additional file 2: Table S1d display a selection of significant GOs from all three categories Biological Process (BP), Cellular Component (CC) and Molecular Function (MF) in MM. For the GO-BPs the terms *biogenesis*, *regulation of nitrogen compound metabolic process, positive regulation of protein metabolic process, response to biotic stimulus, DNA damage response, detection of DNA damage, positive regulation of phospholipid metabolic process*, *blood vessel remodeling, cell cycle arrest, response to unfolded protein, immune response* and *interleukin (IL)-10 production* were significant. These are indicative of the host repair and recovery mechanisms following macromolecular damage and inflammation. Amongst the GO-CCs, the terms *cytoplasm, intracellular, nucleoplasm* and *membrane-bounded organelle* were the most significant. In the GO-MFs, the major terms that came up were *RNA polymerase II distal enhancer sequence-specific DNA binding, oxidized purine DNA binding, mismatch repair complex binding* and *damaged DNA binding,* which are indications of transcriptional processes and DNA repair mechanisms in the host [44].

### Down-regulation of cytoskeletal organization, neutrophil degranulation, aberrant signalling associated GOs are enriched in NCM and CM

Furthermore, the GO terms that are over-represented in NCM and CM (Fig. 2a, b, Additional file 2: Table S1b, c) were evaluated. For GO-BPs, the terms *microtubule organizing center organization, glutathione metabolic process, glutathione derivative biosynthetic process, oxidation–reduction process, DNA damage checkpoint, mitotic cell cycle arrest, reactive oxygen species biosynthetic process, removal of superoxide radicals, detoxification, activation of mitogen-activated protein* kinases *(MAP3Ks) activity, negative regulation of cytoskeleton organization and amyloid-beta clearance* were significant*.* These terms are suggestive of *P. falciparum*-mediated antioxidant synthesis and depletion, cell cycle arrest due to DNA damage, cytoskeletal destabilization and possibly, neurological impairment. With regards to the GO-CCs the terms *intracellular, cytoplasm, membrane-enclosed lumen, cell* and *transcriptional repressor complex* were noteworthy. Significant in the GO-MFs were the terms *superoxide dismutase copper chaperone activity, glutathione transferase activity, class I DNA-(apurinic or apyrimidinic site) endonuclease activity* and *protein kinase activity*. These terms are pointers to host defense mechanisms against *P. falciparum* infection by up-regulating anti-oxidative activities and DNA damage repair. It also signifies a probable increase in protein kinases activity, which may destabilize microtubule organization [45–47].

The GO-BPs that were significant in CM include *signal transduction in absence of ligand, neutrophil degranulation, negative regulation of gene expression, neutrophil activation* and *extrinsic apoptotic signalling pathway.* These terms are indicative of parasite driven abnormal signalling and inflammatory provoked tissue destruction in CM [48, 49]. In the GO-CCs, *tertiary granule membrane, specific granule membrane, intracellular organelle part* and *chromosome, centromeric region* were the most significant terms. The terms highly represented in GO-MF for CM were *oxidoreductase activity, acting on paired donors, with incorporation or reduction of molecular oxygen*, *proximal promoter DNA-binding transcription repressor activity, RNA polymerase II-specific* and *zinc ion binding* which demonstrate host defense against ROS, transcription suppression and the necessity of zinc in binding of the *P. falciparum* to host cells [49].

### Differential expression between CM and NCM

**Fig. 3:**
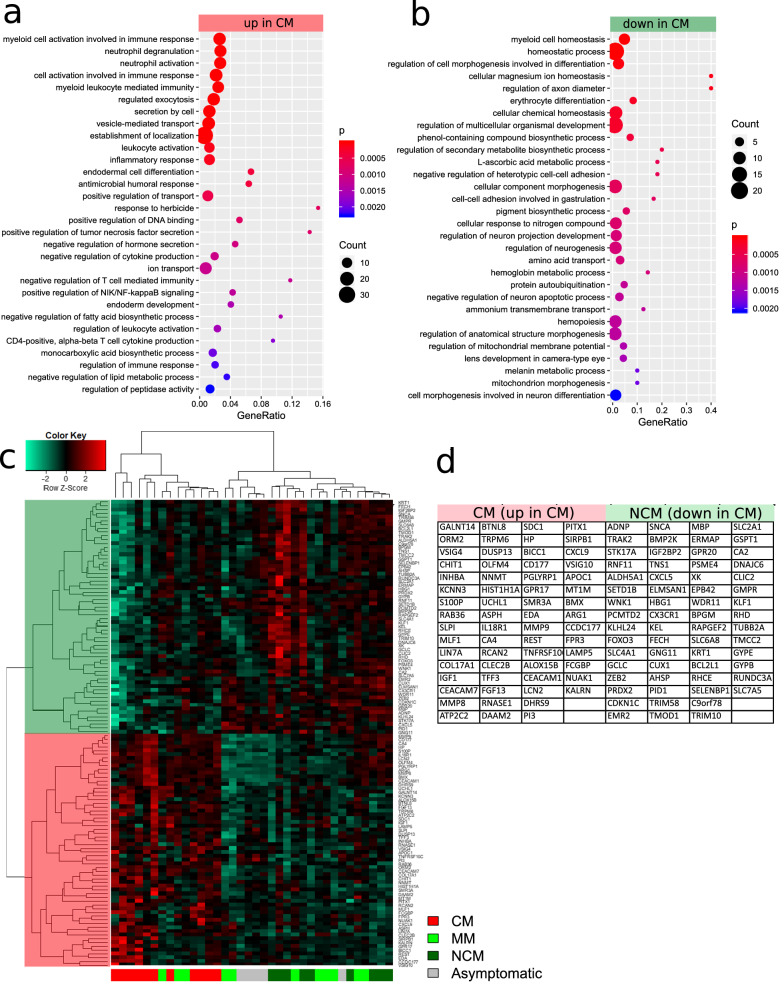
Differential expression analysis between CM and NCM reveals NF-kappaB signalling, immune response, response to herbicide and neutrophil activation amongst GO terms overrepresented in up-regulated genes whereas myeloid cell homeostasis, regulation of axon diameter, haemopoiesis were amongst GO terms highly expressed in downregulated genes. **a** Most significant GO terms elevated in genes up-regulated between CM and NCM. **b** Most significant GO terms elevated in genes down-regulated between CM and NCM. **c** Hierarchical cluster analysis and heatmap of genes up-regulated (ratio > 2, p < 0.05) and down-regulated (ratio < 0.5, p < 0.05) between CM and NCM. The cluster of genes up-regulated in CM is marked with red shading, the cluster of down-regulated genes with green shading. The genes associated with both clusters are listed in **d**. IL18R1 among the up-regulated genes is indicative of the involvement of IL18-signalling

Additionally, the differences between CM and NCM were investigated by further refining the results from the Venn diagram analysis of gene expression. Figure 3 shows signatures of differentially up- and down-regulated genes and associated GOs in CM compared to NCM (Additional file 3: Table S2a). For the genes up-regulated between CM and NCM, NF-kappaB signalling, immune response, response to herbicide and neutrophil activation were amongst the most significantly elevated GO terms (Fig. 3a, Additional file 3: Table S2b). For the genes down-regulated between CM and NCM, myeloid cell homeostasis, regulation of axon diameter, haemopoiesis were amongst the most significantly elevated GO terms (Fig. 3b, Additional file 2: Table S2c). The heatmap and hierarchical cluster analysis in Fig. 3c displays a cluster with higher expression in CM (red) and a cluster with lower expression in CM (green). In Fig. 3d, the signatures of the genes up-regulated (ratio > 2, p < 0.05) and down-regulated (ratio < 0.5, p < 0.05) between CM and NCM are listed. Interestingly, IL-18 receptor 1 (*IL18R1*) is amongst the up-regulated genes indicating the involvement of IL-18 signalling.

### Protein interaction network and regulating transcription factors

**Fig. 4:**
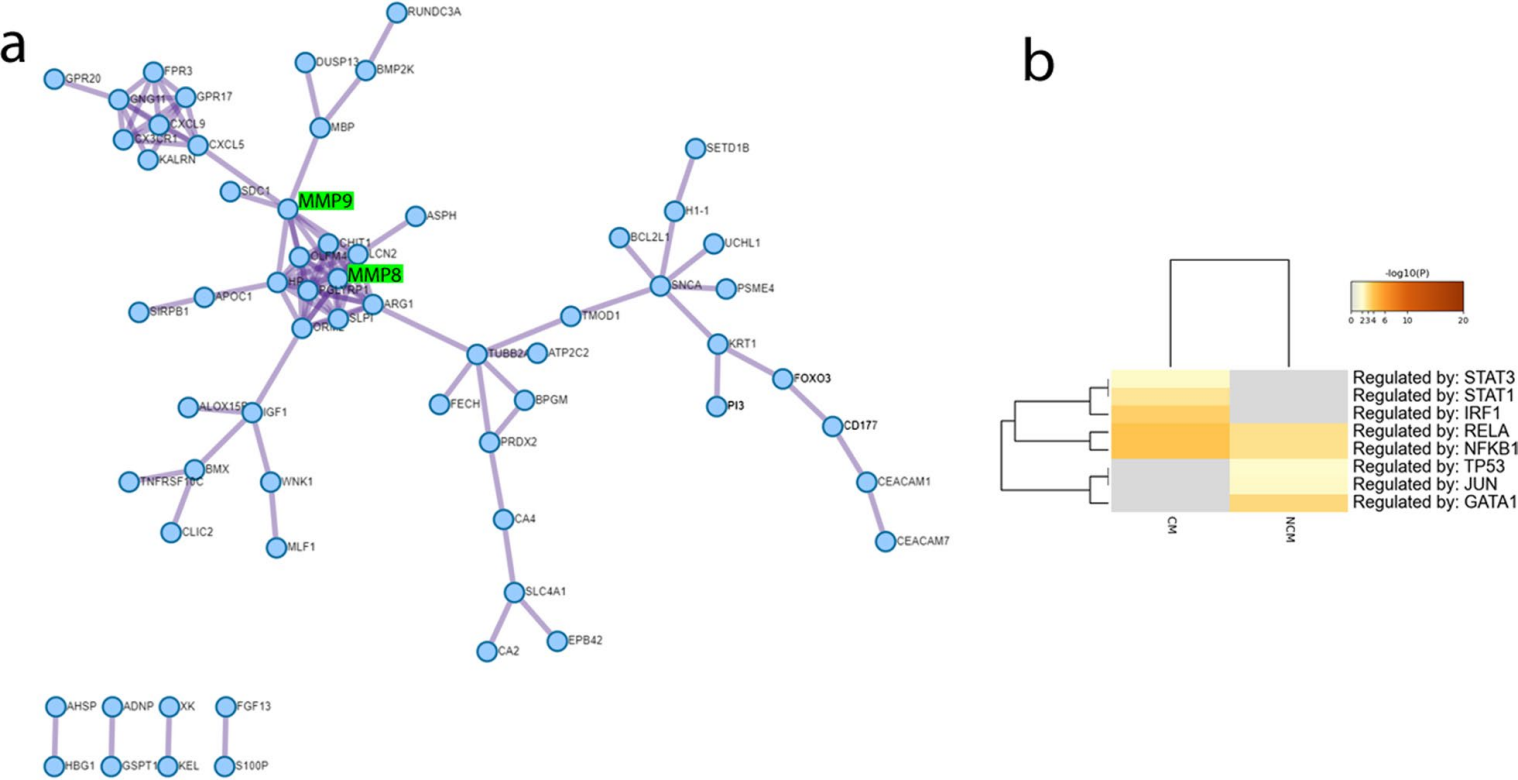
Metascape analysis of genes differentially expressed between CM and NCM reveals protein interaction network and involvement of the transcription factors IRF1, STAT1/3, NFKB1 and RELA in the gene regulatory networks of the CM state. **a** Protein interaction network generated with Metascape from genes differentially expressed between CM and NCM. A central cluster is located around MMP8 and MMP8 which have been associated with CM by Polimeni and Prato, due to their potential to disrupt the subendothelial basement membrane and process and regulate cytokines [16]. Moreover, Polimeni and Prato propose that haemozoin promotes NF-kappaB-controlled gene expression of MMP9. **b** Heatmap of transcription factors generated with Metascape employing the TRRUST method using genes differentially expressed between CM and NCM. Graphics were created with Metascape (https://metascape.org/), [42]

For further elucidation of protein interaction networks and transcription factors involved in gene regulatory networks constituting CM, a Metascape analysis of genes differentially expressed between CM and NCM was employed (Fig. 4). Figure 4a shows the protein interaction network which besides the IL-18 section already mentioned, has a large connected part centered around hexokinase 1 (HK1) and tubulin beta-2A chain (TUBB2A). Figure 4b displays the results of the Metascape TRRUST analysis [43] indicating involvement of the transcription factors, interferon regulatory factor 1 (IRF1), signal transducer and activator of transcription 1/3 (STAT1/3), nuclear factor kappa-B subunit 1 (NFKB1) and RELA proto-oncogene, NF-KB subunit (RELA) in the gene regulatory networks of the CM state.

## Discussion

In this analysis, pooled transcriptome data from whole blood cells of malaria patients, which involved MM, NCM and CM were evaluated. Although transcriptome organization is reported to be poorly preserved between brain and blood, there are some brain co-expression modules which demonstrate strong evidence of preservation in blood and could be useful for identifying blood biomarkers for neurological diseases [50]. Therefore, some extrapolations can be made from transcriptome data from blood (which is more readily accessible) to deduce genes and pathways associated with neurological conditions. Previous investigations by Thiam et al*.* and Boldt et al., which were employed in this analysis had successfully used blood transcriptional analysis to detect changes in gene expression in MM, NCM and CM. In this study, it was discovered from the cluster analysis that the 3 states of *P. falciparum* infection have some common characteristics between them although certain CM states could be distinctly different. While CM was mainly associated with NCM, there was some clustering between MM and CM. Although, there are standards set to define the different states of falciparum malaria, clinical symptoms of the disease can be protean and non-specific without a clear distinction between MM, NCM and CM. This is manifested clinically as fever, malaise, headache, nausea and vomiting, though MM could rapidly progress into CM if not treated quickly [51, 52]. Furthermore, a study by Taylor et al*.* showed that the classification of CM in children is sometimes misdiagnosed, where coma has other causes and parasitaemia is incidental [53]. This suggests that the detection of CM-specific characteristics can be challenging, which could result in its clustering with the other states of falciparum malaria.

In addition, genes that are expressed in the 3-*P. falciparum-*induced conditions were identified. It was discovered that 2876 genes were expressed in common between them and CM surprisingly had the least number of expressed genes. In all, genes associated with host response to parasite invasion, inflammatory response, cellular stress, detoxification of toxic metals and clathrin mediated vesicle assembly were up-regulated. It has been established that entry of malaria parasite into the host elicit immune response, which can lead to oxidative stress [54]. Oxidative stress caused by the release of parasite toxins such as HMZ or inflammatory response to *P. falciparum* entry have been implicated in cellular stress, especially in severe malaria [5, 55]. Moreover, levels of metals such as copper and zinc are increased in acute malaria, which could be as a result of the host’s inflammatory response against parasite and lysis of erythrocytes [56]. Concurrently, there is trafficking of the virulence protein, *P. falciparum* erythrocyte membrane protein 1 (PfEMP1) to the surface of infected red blood cells (iRBCs), which involves membranous organelles termed Maurer’s clefts [57]. Clathrin and its adaptor proteins possibly participate in the process hence its upregulation in the 3 disease states [58]. The genes connected to mental retardation and AD were also present in all 3-forms of falciparum malaria. Previous investigations have documented the predisposition of children with CM to develop mental disorders probably as a result of ischaemic and hypoxic neural injury [59, 60]. The elevation of the expression levels of these genes in MM and NCM implies the possible detrimental nature of these *P. falciparum* infections on the brain as well. Indeed, some studies have documented cognitive deficits in children and adults, who suffered from acute MM and NCM [61–63]. For instance, Hickson et al*.* detected that children with acute kidney injury in NCM developed neurocognitive impairments in both behavioural regulation and executive function [64]. Additionally, Guha et al*.* in using an animal model proved that a single incident of MM induced microglial activation, neuroinflammation and behavioural changes accompanied by increase in pro-inflammatory cytokine expression in the brain [65]. Hence, the severity of neurological deficits reported in CM could be attributed to the down-regulation of various genes involved in cell survival, protein synthesis and mitochondrial respiration. Additionally, a study by Bodlt et al*.* discovered the down-regulation of a number of genes in CM of Gabonese children due to expression of hypoxia-induced genes aryl hydrocarbon receptor (AhRF), GA binding protein transcription factor (GABP) and hypoxia-inducible factor-1 (HIF1) [30]. Brain hypoxia has been documented in CM owing to sequestration of infected erythrocytes, vascular congestion, impaired perfusion and endothelial cell activation [66, 67].

With regards to pathways associated with the different forms of falciparum malaria, KEGG enrichment analysis unveiled the association of MM with the neurodegenerative diseases, PD and PrD. This means MM and some neurodegenerative diseases may share parallel biological processes, which is contradictory to the findings of Thiam et al*.* [28] and Cabantous et al. [29], where neurodegenerative pathways were found in CM only. Nonetheless, processes involved in cancer, EBV infection and DNA damage repair were the most significant. Some human pathogens, including *P. falciparum* and EBV, have been implicated as etiologic agents in the development of cancer [68]. The invasion of *P. falciparum* and subsequent release of HMZ into the host induces inflammation, which facilitates ROS production and cellular stress [69, 70]. ROS production even at low levels can lead to gene and chromosomal mutations via DNA double strand breaks (DSBs), which is one of the most severe forms of DNA damage [71]. DSBs when unrepaired or mis-repaired, can cause stress, cell death, chromosome instability and cancer [72]. EBV infection is also associated with ROS generation [73, 74] and since cancer development can stem from DNA damage, it is not surprising that these pathways were elevated in MM. Again, DNA damage and DNA damage response are hallmarks of neurodegenerative diseases including PD and PrD [75]. The up-regulation of the neurodegenerative pathways in MM suggests a shared cytotoxic mechanism between these diseases and buttresses earlier observation that MM could have implications on brain function.

Similarly, KEGG pathway enrichment assessment was done for NCM and CM. Platinum drug resistance, nucleotide excision repair and glutathione metabolism were associated with NCM. Resistance to anti-malarial drugs is a growing challenge in malarial treatment [76]. The up-regulation of a drug resistance pathway similar to that observed in cancer during NCM implies a parasite facilitated host resistance to anti-malarials. This might be as a result of host cellular modification especially with DNA damage and repair mechanisms, which were also up-regulated in NCM. For instance, multiple efflux and uptake transporters are present in red blood cells and hepatocytes [77, 78]. Increase in and genetically altered drug efflux transporters reduce anti-malarial drug uptake and concentrations in red blood cells [79]. This ensues in minimum effective drug concentration, inhibiting the termination of malaria infection and potentially, the development of resistance. The association of glutathione metabolism in NCM is an indication of glutathione modulation of disease progression particularly with regards to anti-malarial treatment. A study by Zuluaga et al*.* revealed that amodiaquine treatment failure was connected to erythrocytic glutathione [80]. This is because glutathione competes with amodiaquine for the haem group, which could result in therapeutic failure [80]. Again, the concentration of glutathione was decreased in iRBCs, which is an indication of the antioxidant consumption due to ROS generation [81]. Thus, glutathione though an important antioxidant defense in malaria may likely modulate the host’s response to drug treatment. The pathways that correlated with CM were retrograde endocannabinoid signalling and NAFLD. Previous studies have described the up-regulation of certain genes involved in neurodegenerative diseases including amyloid precursor protein (*APP),* ubiquilins *(UBQLN)* and Jun proto-oncogene *(JUN)* as candidate genes for NAFLD [82–85]. In their study, Karbalaei et al*.* showed that there were 190 genes expressed in common between NAFLD and AD, and that NAFLD has an undoubted relation to AD [85]. Moreover, the endocannabinoids and their receptors (especially cannabinoid 1, CB1) are known to induce steatosis and lipogenic gene expression resulting in NAFLD [86, 87]. The cannabinoid 2 (CB2) receptor on the other hand regulates neuro-inflammatory responses and affects various macrophage functions, including antigen uptake and presentation and chemokine/cytokine production [88–91]. A study by Alferink et al*.* reported that mice with a deletion of the CB2-encoding gene (Cnr2^−^/^−^) and immunized with *Plasmodium berghei* (ANKA strain) erythrocytes showed enhanced survival and diminished BBB disruption in experimental CM [88]. Furthermore, the CB1 receptor antagonist rimonabant, is reported to have neuroprotective properties in some animal models of neurodegenerative disorders [92]. In addition, endocannabinoid molecules were significantly increased in the acute phase of *P. falciparum* infection in children [93]. Together, these results reveal that genes involved in NAFLD and the endocannabinoid system could serve as potential biomarkers for malaria severity. This could also be an indication that the consequence of CM on the brain may comprise some neurological pathways other than those classically described for neurodegenerative diseases.

Additionally, the GOs up-regulated in the 3 forms of falciparum malaria were assessed and in MM analysis showed biological processes related to biogenesis, host metabolic processes, DNA damage repair activities and anti-inflammation with the up-regulation of IL-10*.* The imbalance between pro-inflammatory and anti-inflammatory cytokines drives disease severity in falciparum malaria. Boeuf et al*.* observed a high TNF/IL-10 ratio in NCM patients, whereas MM patients had a relatively balanced IL-10 and TNF levels [94]. Thus, in MM the deleterious effect of *P. falciparum* invasion seems to be counteracted by an increase in the host’s metabolic and damage control mechanisms against ROS and inflammatory products.

The GOs elevated in NCM revealed destabilization of cellular cytoskeleton, ROS production, DNA damage checkpoint and amyloid-beta clearance. The surge in ROS levels during oxidative stress is implicated in the increase in the activation of MAP3Ks pathways [95]. An aberration from the strict control of MAP3Ks signalling is connected to the development of many neurodegenerative diseases including AD and PD [96]. Activated MAP3K signalling pathways are thought to contribute to neurodegenerative pathogenesis through the phosphorylation of APP and α-synuclein, leading to aggregates formation that trigger neuronal apoptosis [97–98]. Again, an increase in MAPKs signalling induces cytoskeletal abnormalities through unusual phosphorylation and consequent aggregation of cytoskeletal elements [99–101]. For example, the hyper-phosphorylation of the microtubule associated protein, tau results in its aggregation, microtubule destabilization and the formation of neurofibrillary tangles with subsequent degeneration of neurons [102–105]. Also, axonal injury with the accumulation of APP has been reported in post-mortem studies of CM patients, both adults and paediatric cases [106–108]. In a previous study, brain swelling as a consequence of venous congestion by iRBCs was observed in NCM patients on admission [109], pointing to a possible neuronal injury. Accordingly, the amyloid-beta clearance observed in NCM could represent a reversible pathway to neurological damage in NCM. With respect to CM, abnormal signalling, neutrophil degranulation, repression of gene expression and apoptosis were the predominant GO terms. Using an experimental model of CM, a recent study by Kumar et al*.* reported an increase in D1 and D2 dopaminergic receptor expression, phosphorylated dopamine- and cAMP-regulated phosphoprotein (DARPP), p25, cyclin-dependent kinase 5 (CDK5), Ca^2+^/calmodulin-dependent protein kinase IIα (CaMKIIα) and D1-D2 heteromers [110]. The dysregulation of the dopaminergic receptors led to the impairment and degeneration of medium spiny neurons [110]. Findings from neuroimaging studies have demonstrated that the anatomical and functional grouping of dopaminergic neurons in striatum performs a significant role in the execution of cognition and behavioural consequences in young adults [111, 112]. The cognitive deficit observed in CM survivors therefore could be attributed in part to the dysregulation of the dopaminergic system. Although neutrophils are scarce in the central nervous system under normal physiological conditions, they infiltrate the brain in several pathological conditions, including malaria [113]. The accumulation of these granulocytes lead to the release of neutrophil extracellular traps (NETs) which directly damage the BBB, surrounding neurons and consequently apoptosis [49]. Additionally, Boldt et al*.* in their findings showed a down-regulation of pre-synaptic synuclein alpha (*SNCA*), hydroxyacylglutathione hydrolase (*HAGH*), ankyrin 1 (*ANK1*), ferro chelatase (*FECH*), *STAT1*, homolog A, nucleotide excision repair (*RAD23A*) and 2,3-bisphosphoglycerate mutase (*BPGM*) genes in CM patients [30]. Hence, the up-regulation of genes involved in gene repression is an indicator that several genes involved in cellular metabolism and function are down-regulated in CM, which accounts for the severity of the disease as compared to MM.

Most outstanding of the inflammatory processes found associated with the genes up-regulated in CM were neutrophil activation and IL18-signalling. Previous publications have described IL18 as a major player in the pathogenesis of severe malaria through a pathway of elevating IFN-gamma [114]. The activation of this pathway is further supported by the transcription factor IRF1, for which there was a regulatory role in CM via Metascape analysis. The relevance of neutrophils for inflammatory aggravation of malaria has been described by Knacksted et al*.* [47]. This is mediated by the extrusion of chromatin in the form of NETs upon neutrophil death. In addition, up-regulation of genes linked to herbicide response reiterate the significance of the “plant-like” apicoplast in *P. falciparum* virulence [115]. The GO term *regulation of axon diameter* found in the down-regulated genes in CM provides a mechanistic clue of neurological impairments in CM. The involved genes *XK* and *KEL* were furthermore reported to induce neuropathological abnormalities, such as giant axons when knocked out in mice [116]. The protein interaction network besides relevant sub-networks of IL-18 signalling and the blood group proteins, membrane transport protein X-linked-Kx (XK) and kell metallo-endopeptidase (KEL) regulating axon diameter showed a large connected area centered by TUBB2A and the metalloproteinases 8 and 9 (MMP8, MMP9). TUBB2A has been reported to be up-regulated in CM by Cabantous et al*.* [29] as part of the Parkin-Ubiquitin proteasome degradation pathway, which when impaired can be deleterious to dopaminergic neurons [117]. MMP8 and MMP9 which is activated by HMZ have been associated with CM by Polimeni and Prato due to their potential to disrupt the sub-endothelial basement membrane and process and regulate cytokines [16].

A limitation of this study is that, the number of patients enrolled in the investigations were few and hence larger studies are required to determine the pernicious effect of *P. falciparum* infection on the brain. Furthermore, the study was based only on blood-derived cells and included no brain biopsies, thus implying that some neurological consequences during and after malaria infection are already manifested in the genetic networks in blood-derived cells. However, for the patients included in this study it is not known how many, if any, experienced neurological sequelae subsequent to malaria infection.

## Conclusion

This study has proven that all forms of malaria including MM could have detrimental implications on the brain. The difference in the severity of the various forms of malaria is however dependent on which genes are down/up-regulated in its progression. Also, given the up-regulation of the endocannabinoid and the dysregulation of dopaminergic systems reported in CM, studies into the interplay of CB1/CB2 and D1/D2 receptors and their involvement in neurological sequelae may be conducted. Howbeit, the apicoplast remains a vital target for anti-malarial drugs. It is anticipated that the outcome of this study would form the basis for future hypothesis-driven malaria research.

## Supplementary Information

**Additional file 1: Figure S1**. Distribution of log2-ratios of CM vs. NCM. A Gaussian distribution curve is overlaid (mean=0, sd=0.36). Red lines indicate ratio of 2 (0.5) and blue lines ratio of 1.5 (2/3). The area under curve for ratio 1.5 and 2/3 is ~ 90%, the area for ratio 2 and 0.5 is > 99%.

**Additional file 2: Table S1**. Venn diagram analysis of CM, NCM and MM. (a) subsets of genes in the Venn diagram, (b) GO analysis results from the 77 genes expressed exclusively in CM, (c) GO analysis results from the 289 genes expressed exclusively in NCM, (d) GO analysis results from the 66 genes expressed exclusively in MM, (e) KEGG analysis results from the 77 genes expressed exclusively in CM, (f) KEGG analysis results from the 289 genes expressed exclusively in NCM, (g) KEGG analysis results from the 66 genes expressed exclusively in MM. GO analysis results are sorted by the top GO categories Biological Process (BP), Cellular Component (CC) and Molecular Function (MF) and within these categories by p-value

**Additional file 3: Table S2**. Differential expression analysis of CM vs. NCM. (a) genes significantly up-regulated (ratio > 2, p < 0.05, q < 0.25) and down-regulated (ratio < 0.5, p < 0.05, q < 0.25), (b) GO analysis results from the genes significantly up-regulated in CM, (c) GO analysis results from the genes significantly down-regulated in CM, (d) KEGG pathway analysis results from the genes significantly up-regulated in CM, (e) KEGG pathway analysis results from the genes significantly down-regulated in CM.

## Data Availability

The datasets analysed during the current study are available in the National Center for Biotechnology Information—Gene Expression Omnibus (NCBI GEO) repository under the accessions GSE116306 (https://www.ncbi.nlm.nih.gov/geo/query/acc.cgi?acc=GSE116306) and GSE1124 (https://www.ncbi.nlm.nih.gov/geo/query/acc.cgi?acc=GSE1124). The datasets are summarized in Table 1.

## Abbreviations

AhRF: Aryl hydrocarbon receptor
AD: Alzheimer’s disease
ANK1: Ankyrin 1
APP: Amyloid precursor protein
AUTS2: Autism susceptibility gene 2
BBB: Blood brain barrier
BP: Biological Process
BPGM: 2,3-Bisphosphoglycerate mutase
CaMKIIα: Ca^2^^+^/calmodulin-dependent protein kinase IIα
CB1/CB2: Cannabinoid receptors 1/2
CC: Cellular Component
CDK5: Cyclin-dependent kinase 5
CKLF: Chemokine-like factor
CM: Cerebral malaria
D1/D2: Dopaminergic receptors 1/2
DARPP: Dopamine- and cAMP-regulated phosphoprotein
DNA: Deoxyribonucleic acid
DSBs: Double strand breaks
EBV: Epstein-Barr virus
ER: Endoplasmic reticulum
FECH: Ferro chelatase
GABP: GA binding protein transcription factor
GO: Gene ontology
HAGH: Hydroxyacylglutathione hydrolase
HK1: Hexokinase 1
HMZ: Hemozoin
HIF1: Hypoxia inducible factor-1
iRBCs: Infected red blood cells
IRF1: Interferon regulatory factor 1
IL: Interleukin
JUN: Jun proto-oncogene
KEL: Kell metallo-endopeptidase
KEGG: Kyoto Encyclopedia of Genes and Genomes
MAP3Ks: Mitogen-activated protein kinases
MF: Molecular Function
MM: Mild malaria
MMP8/9: Metalloproteinases 8 and 9
MT1X: Metallothionein-1X
NAFLD: Non-alcoholic fatty liver disease
NCM: Non-cerebral severe malaria
NET: Neutrophil extracellular traps
NFKB1: Nuclear factor kappa-B subunit 1
PD: Parkinson’s disease
PfEMP1: *Plasmodium falciparum* Erythrocyte membrane protein 1
PICALM: Phosphatidylinositol-binding clathrin assembly protein
PrD: Prion disease
PSEN1: Presenilin-1
RAD23A: Homolog A, nucleotide excision repair
RELA: RELA proto-oncogene, NF-KB Subunit
RNA: Ribonucleic acid
RNF-13: Ring finger protein 13
ROS: Reactive oxygen species
STAT1/3: Signal transducer and activator of transcription 1/3
SNCA: Synuclein alpha
TUBB2A: Tubulin beta-2A chain
UBQLN: Ubiquilins
WHO: World Health Organization
XK: Membrane transport protein X-linked-Kx

## References

[1] WHO. World malaria report 2020: 20 years of global progress and challenges. 30.11.2020 edition. Geneva: World Health Organization; 2020. https://www.who.int/publications/i/item/9789240015791.

[2] Mukhtar MM, Eisawi OA, Amanfo SA, Elamin EM, Imam ZS, Osman FM (2019). *Plasmodium vivax* cerebral malaria in an adult patient in Sudan. Malar J.

[3] Eugenin EA, Martiney JA, Berman JW. The malaria toxin hemozoin induces apoptosis in human neurons and astrocytes: potential role in the pathogenesis of cerebral malaria. Brain Res. 2019;1720:146317. 10.1016/j.brainres.2019.146317 PMC6702100 31276637

[4] Imai T, Iwawaki T, Akai R, Suzue K, Hirai M, Taniguchi T (2014). Evaluating experimental cerebral malaria using oxidative stress indicator OKD48 mice. Int J Parasitol.

[5] Kavishe RA, Koenderink JB, Alifrangis M (2017). Oxidative stress in malaria and artemisinin combination therapy: pros and cons. FEBS J.

[6] Simão-Gurge RM, Wunderlich G, Cricco JA, Cubillos EFG, Doménech-Carbó A, Cebrián-Torrejón G (2019). Biosynthesis of heme O in intraerythrocytic stages of *Plasmodium falciparum* and potential inhibitors of this pathway. Sci Rep.

[7] Nagaraj VA, Sundaram B, Varadarajan NM, Subramani PA, Kalappa DM, Ghosh SK, et al. Malaria parasite-synthesized heme is essential in the mosquito and liver stages and complements host heme in the blood stages of infection. PLoS Pathog. 2013;9:e1003522. 10.1371/journal.ppat.1003522 PMC3731253 23935500

[8] Lim L, McFadden GI (2010). The evolution, metabolism and functions of the apicoplast. Philos Trans R Soc Lond B Biol Sci.

[9] Pishchany G, Skaar EP. Taste for blood: hemoglobin as a nutrient source for pathogens. PLoS Pathog. 2012;8:e1002535. 10.1371/journal.ppat.1002535 PMC3297580 22412370

[10] Slater AF, Cerami A (1992). Inhibition by chloroquine of a novel haem polymerase enzyme activity in malaria trophozoites. Nature.

[11] Grau GE, Mackenzie CD, Carr RA, Redard M, Pizzolato G, Allasia C (2003). Platelet accumulation in brain microvessels in fatal pediatric cerebral malaria. J Infect Dis.

[12] Sullivan AD, Ittarat I, Meshnick SR (1996). Patterns of haemozoin accumulation in tissue. Parasitology.

[13] Kwiatkowski D, Bate CA, Scragg IG, Beattie P, Udalova I, Knight JC (1997). The malarial fever response - pathogenesis, polymorphism and prospects for intervention. Ann Trop Med Parasitol.

[14] Sherry BA, Alava G, Tracey KJ, Martiney J, Cerami A, Slater AF (1995). Malaria-specific metabolite hemozoin mediates the release of several potent endogenous pyrogens (TNF, MIP-1 alpha, and MIP-1 beta) in vitro, and altered thermoregulation in vivo. J Inflamm.

[15] Ihekwereme CP, Esimone CO, Nwanegbo EC. Hemozoin inhibition and control of clinical malaria. Adv Pharmacol Sci. 2014;984150. 10.1155/2014/984150 PMC3941158 24669217

[16] Polimeni M, Prato M (2014). Host matrix metalloproteinases in cerebral malaria: new kids on the block against blood-brain barrier integrity?. Fluids Barriers CNS.

[17] Oluwayemi IO, Brown BJ, Oyedeji OA, Oluwayemi MA (2013). Neurological sequelae in survivors of cerebral malaria. Pan Afr Med J.

[18] Bhandary B, Marahatta A, Kim H-R, Chae H-J (2012). An Involvement of oxidative stress in endoplasmic reticulum stress and its associated diseases. Int J Mol Sci.

[19] Hussain T, Tan B, Yin Y, Blachier F, Tossou MCB, Rahu N (2016). Oxidative stress and inflammation: what polyphenols can do for us?. Oxid Med Cell Longev.

[20] Muthuswamy AD, Vedagiri K, Ganesan M, Chinnakannu P (2006). Oxidative stress-mediated macromolecular damage and dwindle in antioxidant status in aged rat brain regions: role of L-carnitine and DL-alpha-lipoic acid. Clin Chim Acta.

[21] Kannan K, Jain SK (2000). Oxidative stress and apoptosis. Pathophysiology.

[22] Redza-Dutordoir M, Averill-Bates DA (2016). Activation of apoptosis signalling pathways by reactive oxygen species. Biochim Biophys Acta.

[23] Hegde ML, Hegde PM, Rao KS, Mitra S (2011). Oxidative genome damage and its repair in neurodegenerative diseases: function of transition metals as a double-edged sword. J Alzheimers Dis.

[24] Poetsch AR (2020). The genomics of oxidative DNA damage, repair, and resulting mutagenesis. Comput Struct Biotechnol J.

[25] Sertan Copoglu U, Virit O, Hanifi Kokacya M, Orkmez M, Bulbul F, Binnur Erbagci A (2015). Increased oxidative stress and oxidative DNA damage in non-remission schizophrenia patients. Psychiatry Res.

[26] Singh A, Kukreti R, Saso L, Kukreti S (2019). Oxidative stress: a key modulator in neurodegenerative diseases. Molecules.

[27] Li J, Shang Y, Wang L, Zhao B, Sun C, Li J, et al. Genome integrity and neurogenesis of postnatal hippocampal neural stem/progenitor cells require a unique regulator Filia. Sci Adv. 2020;6:eaba0682. 10.1126/sciadv.aba0682 PMC7608785 33115731

[28] Thiam A, Sanka M, Ndiaye Diallo R, Torres M, Mbengue B, Nunez NF (2019). Gene expression profiling in blood from cerebral malaria patients and mild malaria patients living in Senegal. BMC Med Genomics.

[29] Cabantous S, Poudiougou B, Bergon A, Barry A, Oumar AA, Traore AM (2020). Understanding human cerebral malaria through a blood transcriptomic signature: evidences for erythrocyte alteration, immune/inflammatory dysregulation, and brain dysfunction. Mediators Inflamm.

[30] Boldt ABW, van Tong H, Grobusch MP, Kalmbach Y, Dzeing Ella A, Kombila M (2019). The blood transcriptome of childhood malaria. EBioMedicine.

[31] Gentleman RC, Carey VJ, Bates DM, Bolstad B, Dettling M, Dudoit S (2004). Bioconductor: open software development for computational biology and bioinformatics. Genome Biol.

[32] Smyth GK (2004). Linear models and empirical bayes methods for assessing differential expression in microarray experiments. Stat Appl Genet Mol Biol.

[33] Gautier L, Cope L, Bolstad BM, Irizarry RA (2004). affy–analysis of Affymetrix GeneChip data at the probe level. Bioinformatics.

[34] Johnson WE, Li C, Rabinovic A (2007). Adjusting batch effects in microarray expression data using empirical Bayes methods. Biostatistics.

[35] Leek JT, Johnson WE, Parker HS, Jaffe AE, Storey JD (2012). The sva package for removing batch effects and other unwanted variation in high-throughput experiments. Bioinformatics.

[36] Storey JD (2002). A direct approach to false discovery rates. J R Stat Soc Series B Stat Methodol.

[37] Warnes GR, Bolker B, Bonebakker L, Gentleman R, Liaw WHA, Lumley T, et al. Gplots: various R programming tools for plotting data. 2016. R package version 2014, 2.

[38] Chen H, Boutros PC (2011). VennDiagram: a package for the generation of highly-customizable Venn and Euler diagrams in R. BMC Bioinformatics.

[39] Kanehisa M, Furumichi M, Tanabe M, Sato Y, Morishima K (2017). KEGG: new perspectives on genomes, pathways, diseases and drugs. Nucleic Acids Res.

[40] Falcon S, Gentleman R (2007). Using GOstats to test gene lists for GO term association. Bioinformatics.

[41] Wickham H (2009). ggplot2: Elegant Graphics for Data Analysis.

[42] Zhou Y, Zhou B, Pache L, Chang M, Khodabakhshi AH, Tanaseichuk O (2019). Metascape provides a biologist-oriented resource for the analysis of systems-level datasets. Nat Commun.

[43] Han H, Cho JW, Lee S, Yun A, Kim H, Bae D (2018). TRRUST v2: an expanded reference database of human and mouse transcriptional regulatory interactions. Nucleic Acids Res.

[44] Sampaio NG, Emery SJ, Garnham AL, Tan QY, Sisquella X, Pimentel MA, et al. Extracellular vesicles from early stage *Plasmodium falciparum*-infected red blood cells contain PfEMP1 and induce transcriptional changes in human monocytes. Cell Microbiol. 2018;20:e12822. 10.1111/cmi.12822 29349926

[45] Cavallini A, Brewerton S, Bell A, Sargent S, Glover S, Hardy C (2013). An unbiased approach to identifying tau kinases that phosphorylate tau at sites associated with Alzheimer disease. J Biol Chem.

[46] Morshed N, Lee M, Rodriguez FH, Lauffenburger DA, Mastroeni D, White F (2021). Quantitative phosphoproteomics uncovers dysregulated kinase networks in Alzheimer’s disease. Nat Aging.

[47] Knackstedt SL, Georgiadou A, Apel F, Abu-Abed U, Moxon CA, Cunnington AJ, et al. Neutrophil extracellular traps drive inflammatory pathogenesis in malaria. Sci Immunol. 2019;4:eaaw0336. 10.1126/sciimmunol.aaw0336 PMC6892640 31628160

[48] Boeltz S, Muñoz LE, Fuchs TA, Herrmann M (2017). Neutrophil extracellular traps open the Pandora's Box in severe malaria. Front Immunol.

[49] Marvin RG, Wolford JL, Kidd MJ, Murphy S, Ward J, Que EL (2012). Fluxes in "free" and total zinc are essential for progression of intraerythrocytic stages of *Plasmodium falciparum*. Chem Biol.

[50] Cai C, Langfelder P, Fuller TF, Oldham MC, Luo R, van den Berg LH (2010). Is human blood a good surrogate for brain tissue in transcriptional studies?. BMC Genomics.

[51] Bartoloni A, Zammarchi L. Clinical aspects of uncomplicated and severe malaria. Mediterr J Hematol Infect Dis. 2012;4:e2012026. 10.4084/MJHID.2012.026 PMC3375727 22708041

[52] Marrelli MT, Brotto M (2016). The effect of malaria and anti-malarial drugs on skeletal and cardiac muscles. Malar J.

[53] Taylor TE, Fu WJ, Carr RA, Whitten RO, Mueller JS, Fosiko NG (2004). Differentiating the pathologies of cerebral malaria by postmortem parasite counts. Nat Med.

[54] Long CA, Zavala F. Immune responses in malaria. Cold Spring Harb Perspect Med. 2017;7:a025577. 10.1101/cshperspect.a025577 PMC5538407 28389518

[55] Galluzzi L, Diotallevi A, Magnani M. Endoplasmic reticulum stress and unfolded protein response in infection by intracellular parasites. Future Sci OA. 2017;3:FSO198. 10.4155/fsoa-2017-0020 PMC5583660 28883998

[56] Narsaria N, Mohanty C, Das BK, Mishra SP, Prasad R (2011). Oxidative stress in children with severe malaria. J Trop Pediatr.

[57] McHugh E, Carmo OMS, Blanch A, Looker O, Liu B, Tiash S, et al. Role of *Plasmodium falciparum* protein GEXP07 in Maurer's cleft morphology, knob architecture, and *P. falciparum* EMP1 trafficking. mBio. 2020;11:e03320–19. 10.1128/mBio.03320-19 PMC7078486 32184257

[58] Taraschi TF, Trelka D, Martinez S, Schneider T, O'Donnell ME (2001). Vesicle-mediated trafficking of parasite proteins to the host cell cytosol and erythrocyte surface membrane in *Plasmodium falciparum* infected erythrocytes. Int J Parasitol.

[59] Idro R, Kakooza-Mwesige A, Asea B, Ssebyala K, Bangirana P, Opoka RO (2016). Cerebral malaria is associated with long-term mental health disorders: a cross sectional survey of a long-term cohort. Malar J.

[60] Jain K, Sood S, Gowthamarajan K (2013). Modulation of cerebral malaria by curcumin as an adjunctive therapy. Braz J Infect Dis.

[61] Fernando SD, Rodrigo C, Rajapakse S (2010). The 'hidden' burden of malaria: cognitive impairment following infection. Malar J.

[62] Holding PA, Snow RW (2001). Impact of *Plasmodium falciparum* malaria on performance and learning: review of the evidence. Am J Trop Med Hyg.

[63] Kihara M, Carter JA, Newton CR (2006). The effect of *Plasmodium falciparum* on cognition: a systematic review. Trop Med Int Health.

[64] Hickson MR, Conroy AL, Bangirana P, Opoka RO, Idro R, Ssenkusu JM, et al. Acute kidney injury in Ugandan children with severe malaria is associated with long-term behavioral problems. PLoS One. 2019;14:e0226405. 10.1371/journal.pone.0226405 PMC6917349 31846479

[65] Guha SK, Tillu R, Sood A, Patgaonkar M, Nanavaty IN, Sengupta A (2014). Single episode of mild murine malaria induces neuroinflammation, alters microglial profile, impairs adult neurogenesis, and causes deficits in social and anxiety-like behavior. Brain Behav Immun.

[66] Yusuf FH, Hafiz MY, Shoaib M, Ahmed SA (2017). Cerebral malaria: insight into pathogenesis, complications and molecular biomarkers. Infect Drug Resist.

[67] Seydel KB, Kampondeni SD, Valim C, Potchen MJ, Milner DA, Muwalo FW (2015). Brain swelling and death in children with cerebral malaria. N Engl J Med.

[68] Robbiani Davide F, Deroubaix S, Feldhahn N, Oliveira Thiago Y, Callen E, Wang Q (2015). *Plasmodium* infection promotes genomic instability and AID-dependent B- cell lymphoma. Cell.

[69] Percário S, Moreira DR, Gomes BA, Ferreira ME, Gonçalves AC, Laurindo PS (2012). Oxidative stress in malaria. Int J Mol Sci.

[70] Keller CC, Kremsner PG, Hittner JB, Misukonis MA, Weinberg JB, Perkins DJ (2004). Elevated nitric oxide production in children with malarial anemia: hemozoin-induced nitric oxide synthase type 2 transcripts and nitric oxide in blood mononuclear cells. Infect Immun.

[71] Sharma V, Collins LB, Chen T-H, Herr N, Takeda S, Sun W (2016). Oxidative stress at low levels can induce clustered DNA lesions leading to NHEJ mediated mutations. Oncotarget.

[72] Trenner A, Sartori AA (2019). Harnessing DNA double-strand break repair for cancer treatment. Front Oncol.

[73] Huang S-Y, Fang C-Y, Wu C-C, Tsai C-H, Lin S-F, Chen J-Y. Reactive oxygen species mediate epstein-barr virus reactivation by N-Methyl-N’-Nitro-N-Nitrosoguanidine. PLoS One. 2013;8:e84919. 10.1371/journal.pone.0084919 PMC3869928 24376853

[74] Lassoued S, Ben Ameur R, Ayadi W, Gargouri B, Ben Mansour R, Attia H (2008). Epstein-Barr virus induces an oxidative stress during the early stages of infection in B lymphocytes, epithelial, and lymphoblastoid cell lines. Mol Cell Biochem.

[75] Bujdoso R, Landgraf M, Jackson WS, Thackray AM (2015). Prion-induced neurotoxicity: possible role for cell cycle activity and DNA damage response. World J Virol.

[76] Romphosri S, Changruenngam S, Chookajorn T, Modchang C (2020). Role of a concentration gradient in malaria drug resistance evolution: a combined within- and between-hosts modelling approach. Sci Rep.

[77] Nies AT (2007). The role of membrane transporters in drug delivery to brain tumors. Cancer Lett.

[78] Nies AT, Schwab M, Keppler D (2008). Interplay of conjugating enzymes with OATP uptake transporters and ABCC/MRP efflux pumps in the elimination of drugs. Expert Opin Drug Metab Toxicol.

[79] Kerb R, Fux R, Mörike K, Kremsner PG, Gil JP, Gleiter CH (2009). Pharmacogenetics of antimalarial drugs: effect on metabolism and transport. Lancet Infect Dis.

[80] Zuluaga L, Pabón A, López C, Ochoa A, Blair S (2007). Amodiaquine failure associated with erythrocytic glutathione in *Plasmodium falciparum* malaria. Malar J.

[81] Ginsburg H, Golenser J (2003). Glutathione is involved in the antimalarial action of chloroquine and its modulation affects drug sensitivity of human and murine species of *Plasmodium*. Redox Rep.

[82] Daoud H, Rouleau GA (2011). A role for ubiquilin 2 mutations in neurodegeneration. Nat Rev Neurol.

[83] Qi S, Wang C, Li C, Wang P, Liu M. Candidate genes investigation for severe nonalcoholic fatty liver disease based on bioinformatics analysis. Medicine 2017; 96:e7743. 10.1097/MD.0000000000007743 PMC5556226 28796060

[84] Wu M, Fang K, Wang W, Lin W, Guo L, Wang J (2019). Identification of key genes and pathways for Alzheimer’s disease via combined analysis of genome-wide expression profiling in the hippocampus. Biophys Rep.

[85] Karbalaei R, Allahyari M, Rezaei-Tavirani M, Asadzadeh-Aghdaei H, Zali MR (2018). Protein-protein interaction analysis of Alzheimers disease and NAFLD based on systems biology methods unhide common ancestor pathways. Gastroenterol Hepatol Bed Bench.

[86] Schwabe RF (2005). Endocannabinoids promote hepatic lipogenesis and steatosis through CB1 receptors. Hepatology.

[87] Jeong WI, Osei-Hyiaman D, Park O, Liu J, Bátkai S, Mukhopadhyay P (2008). Paracrine activation of hepatic CB1 receptors by stellate cell-derived endocannabinoids mediates alcoholic fatty liver. Cell Metab.

[88] Alferink J, Specht S, Arends H, Schumak B, Schmidt K, Ruland C (2016). Cannabinoid receptor 2 modulates susceptibility to experimental cerebral malaria through a CCL17-dependent mechanism. J Biol Chem.

[89] Cabral GA, Griffin-Thomas L: Emerging role of the cannabinoid receptor CB2 in immune regulation: therapeutic prospects for neuroinflammation. Expert Rev Mol Med. 2009;11:e3. 10.1017/S1462399409000957 PMC2768535 19152719

[90] Centonze D, Rossi S, Finazzi-Agrò A, Bernardi G, Maccarrone M (2007). The (endo)cannabinoid system in multiple sclerosis and amyotrophic lateral sclerosis. Int Rev Neurobiol.

[91] Rossi S, Bernardi G, Centonze D (2010). The endocannabinoid system in the inflammatory and neurodegenerative processes of multiple sclerosis and of amyotrophic lateral sclerosis. Exp Neurol.

[92] Fowler CJ, Rojo ML, Rodriguez-Gaztelumendi A (2010). Modulation of the endocannabinoid system: neuroprotection or neurotoxicity?. Exp Neurol.

[93] Surowiec I, Gouveia-Figueira S, Orikiiriza J, Lindquist E, Bonde M, Magambo J (2017). The oxylipin and endocannabidome responses in acute phase *Plasmodium falciparum* malaria in children. Malar J.

[94] Boeuf PS, Loizon S, Awandare GA, Tetteh JKA, Addae MM, Adjei GO (2012). Insights into deregulated TNF and IL-10 production in malaria: implications for understanding severe malarial anaemia. Malar J.

[95] Son Y, Cheong Y-K, Kim N-H, Chung H-T, Kang DG, Pae H-O. Mitogen-activated protein kinases and reactive oxygen species: How can ros activate MAPK pathways? J Signal Transduct. 2011;2011:792639. 10.1155/2011/792639 PMC3100083 21637379

[96] Puig B, Gómez-Isla T, Ribe E, Cuadrado M, Torrejón-Escribano B, Dalfo E (2004). Expression of stress-activated kinases c-Jun N-terminal kinase (SAPK/JNK-P) and p38 kinase (p38-P), and tau hyperphosphorylation in neurites surrounding βA plaques in APP Tg2576 mice. Neuropathol Appl Neurobiol.

[97] Chiarini A, Pra ID, Marconi M, Chakravarthy B, Whitfield JF, Armato U (2009). Calcium-sensing receptor (CaSR) in human brain's pathophysiology: roles in late-onset Alzheimer's disease (LOAD). Curr Pharm Biotechnol.

[98] Hashimoto Y, Tsuji O, Niikura T, Yamagishi Y, Ishizaka M, Kawasumi M (2003). Involvement of c-Jun N-terminal kinase in amyloid precursor protein-mediated neuronal cell death. J Neurochem.

[99] Brownlees J, Yates A, Bajaj N, Davis D, Anderton B, Leigh P (2000). Phosphorylation of neurofilament heavy chain side-arms by stress activated protein kinase-1b/Jun N-terminal kinase-3. J Cell Sci.

[100] Ackerley S, Grierson AJ, Banner S, Perkinton MS, Brownlees J, Byers HL (2004). p38α stress-activated protein kinase phosphorylates neurofilaments and is associated with neurofilament pathology in amyotrophic lateral sclerosis. Mol Cell Neurosci.

[101] Kim EK, Choi E-J (2010). Pathological roles of MAPK signaling pathways in human diseases. Biochim Biophys Acta.

[102] Wang J-Z, Liu F (2008). Microtubule-associated protein tau in development, degeneration and protection of neurons. Prog Neurobiol.

[103] Pérez M, Morán MA, Ferrer I, Ávila J, Gómez-Ramos P (2008). Phosphorylated tau in neuritic plaques of APP sw/Tau vlw transgenic mice and Alzheimer disease. Acta Neuropathol.

[104] Eckermann K, Mocanu M-M, Khlistunova I, Biernat J, Nissen A, Hofmann A (2007). The β-propensity of Tau determines aggregation and synaptic loss in inducible mouse models of tauopathy. J Biol Chem.

[105] Alonso AD, Cohen LS, Corbo C, Morozova V, ElIdrissi A, Phillips G (2018). Hyperphosphorylation of Tau associates with changes in its function beyond microtubule stability. Front Cell Neurosci.

[106] Dorovini-Zis K, Schmidt K, Huynh H, Fu W, Whitten RO, Milner D (2011). The neuropathology of fatal cerebral malaria in malawian children. Am J Pathol.

[107] Severe malaria. Trop Med Int Health. 2014;19 Suppl 1:7–131. 10.1111/tmi.12313_2 25214480

[108] Medana IM, Day NP, Hien TT, Mai NT, Bethell D, Phu NH (2002). Axonal injury in cerebral malaria. Am J Pathol.

[109] Maude RJ, Barkhof F, Hassan MU, Ghose A, Hossain A, Abul Faiz M (2014). Magnetic resonance imaging of the brain in adults with severe falciparum malaria. Malar J.

[110] Kumar SP, Babu PP. Aberrant dopamine receptor signaling plays critical role in the impairment of striatal neurons in experimental cerebral malaria. Mol Neurobiol. 2020;57:5069–83. 10.1007/s12035-020-02076-0 32833186

[111] Aarts E, van Holstein M, Cools R (2011). Striatal dopamine and the interface between motivation and cognition. Front Psychol.

[112] Provost J-S, Hanganu A, Monchi O (2015). Neuroimaging studies of the striatum in cognition Part I: healthy individuals. Front Syst Neurosci.

[113] Manda-Handzlik A, Demkow U (2019). The brain entangled: the contribution of neutrophil extracellular traps to the diseases of the central nervous system. Cells.

[114] Kojima S, Nagamine Y, Hayano M, Looareesuwan S, Nakanishi K (2004). A potential role of interleukin 18 in severe *falciparum* malaria. Acta Trop.

[115] Ponts N, Yang J, Chung D-WD, Prudhomme J, Girke T, Horrocks P, et al. Deciphering the ubiquitin-mediated pathway in apicomplexan parasites: a potential strategy to interfere with parasite virulence. PLoS One. 2008;3:e2386. 10.1371/journal.pone.0002386 PMC2408969 18545708

[116] Zhu X, Cho ES, Sha Q, Peng J, Oksov Y, Kam SY (2014). Giant axon formation in mice lacking Kell, XK, or Kell and XK: animal models of McLeod neuroacanthocytosis syndrome. Am J Pathol.

[117] Tanaka K, Suzuki T, Hattori N, Mizuno Y (2004). Ubiquitin, proteasome and parkin. Biochim Biophys Acta.

